# Line blot immunoassays in idiopathic inflammatory myopathies: retrospective review of diagnostic accuracy and factors predicting true positive results

**DOI:** 10.1186/s41927-020-00132-9

**Published:** 2020-07-20

**Authors:** Fergus To, Clara Ventín-Rodríguez, Shuayb Elkhalifa, James B. Lilleker, Hector Chinoy

**Affiliations:** 1 Division of Rheumatology, University of British Columbia Division of Rheumatology, University of British Columbia, 802 - 1200 Burrard Street, Vancouver, BC V6Z 2C7 Canada; 2 Complejo Hospitalario Universitario de A Coruña, Xubias de Arriba, 84, 15006 A Coruña, Spain; 3 Department of Immunology, Salford Royal NHS Foundation Trust, 2nd floor, Turnberg Building, Stott Lane, Salford, M68HD UK; 4 Centre for Musculoskeletal Research, School of Biological Sciences, Faculty of Biology, Medicine and Health, Manchester Academic Health Science Centre, The University of Manchester, Stopford Building, 99 Oxford Rd, Manchester, M13 9PG UK; 5 Manchester Centre for Clinical Neurosciences, Salford Royal NHS Foundation Trust, Stott Lane, Salford, M68HD UK; 6 National Institute for Health Research Manchester Biomedical Research Centre, Manchester University NHS Foundation Trust, The University of Manchester, Manchester, UK; 7 Department of Rheumatology, Salford Royal NHS Foundation Trust, Manchester Academic Health Science Centre, Stott Lane, Salford, M68HD UK; 8 Centre for Musculoskeletal Research, Manchester Academic Health Science Centre, The University of Manchester, Manchester, UK

**Keywords:** Immunoblotting; myositis; autoantibodies, Inflammatory muscle disease, Idiopathic inflammatory myopathy, Line blot immunoassay

## Abstract

**Background:**

Line blot immunoassays (LIA) for myositis-specific (MSA) and myositis-associated (MAA) autoantibodies have become commercially available. In the largest study of this kind, we evaluated the clinical performance of a widely used LIA for MSAs and MAAs.

**Methods:**

Adults tested for MSA/MAA by LIA at a tertiary myositis centre (January 2016–July 2018) were identified. According to expert-defined diagnoses, true and false positive rates were calculated for strongly and weakly positive autoantibody results within three cohorts: idiopathic inflammatory myopathy (IIM), connective tissue disease (CTD) without myositis, and non-CTD/IIM. Factors associated with true positivity were determined.

**Results:**

We analysed 342 cases. 67 (19.6%) had IIM, in whom 71 autoantibodies were detected (50 strong positives [70.4%], 21 weak positives [29.6%]). Of the strong positives, 48/50 (96.0%; 19 MSAs, 29 MAAs) were deemed true positives. Of the weak positives, 15/21 (71.4%; 3 MSAs, 12 MAAs) were deemed true positives.

In CTD without myositis cases (*n =* 120), 31/61 (51.0%; 5 MSAs, 26 MAAs) autoantibodies were strongly positive, with 24/31 (77.4%; 0 MSAs, 24 MAAs) true positives. 30/61 (49.2%; 13 MSAs, 17 MAAs) were weakly positive, with 16/30 (53.3%; 0 MSAs, 16 MAAs) true positives. In non-CTD/IIM cases (*n* = 155), all 24 MSAs and 22 MAAs were false positives; these results included 17 (37.0%; 7 MSAs, 10 MAAs) strong positives.

Individual autoantibody specificities were > 98.2 and > 97.5% for weakly and strongly positive results, respectively. True positivity was associated with high pre-test for IIM (odds ratio 50.8, 95% CI 13.7–189.2, *p* < 0.001) and strong positive (versus weak positive) results (4.4, 2.3–8.3, *p* < 0.001).

**Conclusions:**

We demonstrated the high specificity of a myositis LIA in a clinical setting. However, a significant burden of false positive results was evident in those with a low pre-test likelihood of IIM and for weakly positive autoantibodies.

## Background

The idiopathic inflammatory myopathies (IIM) are heterogeneous multisystem autoimmune diseases often presenting with skeletal muscle weakness, rash, arthritis, and/or interstitial lung disease [1]. IIM subtypes include immune-mediated necrotising myopathy (IMNM), sporadic inclusion-body myositis (IBM), overlap myositis (OM), anti-synthetase syndrome (ASS), dermatomyositis (DM) and polymyositis (PM) [1–5].

Approximately 2/3 of IIM patients have detectable serum autoantibodies (Abs), termed myositis-specific autoantibodies (MSA), which are unique to IIM and usually mutually exclusive to one another, or myositis-associated autoantibodies (MAA) which can occur in other connective tissue diseases (CTD) [6–8]. MSA/MAAs are gaining importance in IIM diagnosis, can circumvent the requirement for a muscle biopsy, and may inform prognosis [9–18].

Line blot immunoassays (LIA) for MSA/MAA have become commercially available, increasing the availability of testing in clinical practice [19]. Literature suggests LIA is an appropriate substitute to conventional immunoprecipitation for MSA/MAA testing, but only small samples have been studied with variable accuracy demonstrated [13, 19, 20].

In the largest study of its kind, we evaluated the diagnostic accuracy of a commercially available LIA for MSA and MAA testing in a clinical setting and examined factors associated with true positive results.

## Methods

### Cases

We retrospectively identified patients tested with the EUROLINE Inflammatory Myopathies 16 Ag (IgG) commercial LIA (Euroimmun, Lubeck, Germany) from January 1st, 2016, to July 30th, 2018, at Salford Royal NHS Foundation Trust (SRFT), United Kingdom. The search was limited to patients being reviewed in tertiary IIM, systemic sclerosis and neuromuscular outpatient clinics. Patients without available clinical data were removed. Where duplicate testing occurred, only the most recent results were analysed.

This study was performed as part of a quality improvement project evaluating LIA usage at SRFT. Case notes and other data were reviewed retrospectively without alteration to patient management. Given this context, and after consultation with the Health Research Authority (via www.hra-decisiontools.org.uk) this study proceeded without further requirement for ethical authorization.

### Autoantibody testing

The studied LIA can detect 12 MSAs (anti-Mi2A, anti-Mi2B, anti-TIF1*γ*, anti-MDA5, anti-NXP2, anti-SAE1, anti-SRP, anti-Jo1, anti-PL7, anti-PL12, anti-EJ, anti-OJ) and 4 MAAs (anti-Ku, anti-PM-Scl100, anti-PM-Scl75, anti-Ro52). Laboratory testing was performed using the scanner, software, and protocol provided by the manufacturer. Results were categorised as negative, weakly positive, or strongly positive according to the signal intensity measured digitally as per the manufacturer’s specifications.

### Clinical data collection methodology

Patient records were reviewed by 2 authors (FT and CR) independently. Demographics, electromyography (EMG) results, muscle biopsy results, and peak serum total creatine kinase (CK) levels were collated. Indications for ordering the LIA (pre-test diagnoses) were categorised retrospectively as “suspected IIM”, “CTD without evidence of myositis”, or “myopathic syndromes with low likelihood of IIM”. The final diagnosis made by the expert treating clinician was recorded and categorised retrospectively as “IIM” (including overlap syndromes), “CTD without myositis”, or “non-IIM/CTD”. Categorisation was agreed on between the authors (FT, CR), and a third author was consulted in indeterminate cases (JBL or HC). Diagnoses were verified through review of extensive clinical information reflecting several years of care in each case. Research classification criteria were not applied as by their nature these are restrictive and would limit the real-world applicability of this study. In addition, recent studies have demonstrated classification criteria may not accurately reflect clinical diagnoses made by expert clinicians [21].

### Assay performance

Ab results were reviewed and categorised as true or false positive according to the available clinical information. True positive MSAs were defined as those in patients diagnosed with IIM with the phenotype and IIM subtype expected of that MSA. True positive MAAs were defined as those in patients diagnosed with either CTD or IIM phenotypes expected of that MAA. Otherwise, results were deemed to be false positives. For patients with multiple MSAs, that which best reflected the clinical phenotype was assigned true positive, as MSAs are generally mutually exclusive [8]. Additional MSAs in such cases were false positives, except for anti-Mi2A and anti-Mi2B (isoforms of the same Ab) where simultaneous true positives were accepted [22, 23]. All negative results were deemed true negatives. In cases of uncertainty, an immunologist (SE) reviewed the source data to ensure accuracy.

### Statistics

Analysis was performed with STATA version 14 (College Station, USA). Descriptive statistics examined characteristics of different groups according to Ab status. Categorical data were summarised as frequencies and proportions. Continuous data were summarised using means and standard deviations. For individual Abs, the rate of true and false positivity and the associated specificity for the presence of a consistent diagnosis or disease subtype was calculated. Logistic regression was performed to investigate factors associated with true positive results. A *p*-value < 0.05 represented a statistically significant difference.

## Results

### Patient characteristics

**Fig. 1:**
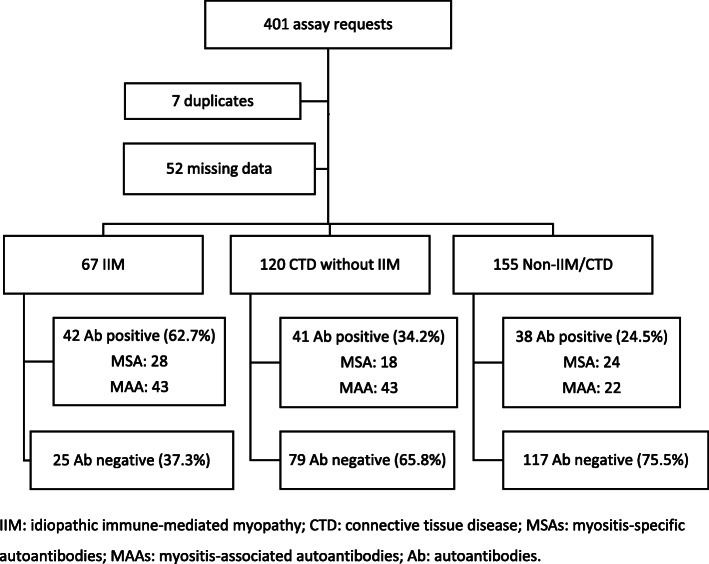
Study population categorised by final diagnoses and total number of Abs detected in each group

In total, 401 LIAs were performed in 394 patients. Clinical data were missing for 55 cases and duplicate testing occurred in 7 patients (Fig. 1). Of the remaining 342, IIM was diagnosed in 67/342 (19.6%) patients, CTD without myositis in 120/342 (35.1%) patients, and non-CTD/IIM in 155/342 (45.3%) patients (Table 1).

**Table 1:** Characteristics of study population

Number of patients	
Total	342
Mean age in years (SD)	54 (14)
Female gender	222 (65%)
Pre-test working diagnosis	
Suspected IIM	92 (26.9%)
CTDs without IIM	137 (40.1%)
Myopathic syndromes with low likelihood of IIM	113 (33.0%)
Final diagnosis and subtype	
IIM	67 (19.6%)
Overlap myositis	21
Dermatomyositis	12
Polymyositis	11
Antisynthetase syndrome	7
Amyopathic dermatomyositis	7
Inclusion body myositis	5
Immune mediated necrotizing myopathy	4
CTD without IIM	120 (35.1%)
Systemic sclerosis	91
Undifferentiated CTD	10
Systemic lupus erythematosus	7
Inflammatory arthritis	6
Overlap CTD	4
Sjogrens syndrome	2
Non-IIM/CTD	155 (45.3%)
Other rheumatologic diagnoses	45
Other neurologic diagnoses	12
Genetic myopathy	11
Endocrinologic myopathy	5
Idiopathic pulmonary fibrosis	3
Post-viral myopathy	3
Traumatic myopathy	3
Familial amyloidosis	1
Orbital myositis	1
Malignancy	1
Unclear	70

*SD* standard deviation, *IIM* idiopathic inflammatory myopathy, *CTD* connective tissue disease

### Autoantibody profiles in IIM patients: *Most weak positive MSAs were false positives*

**Fig. 2:**
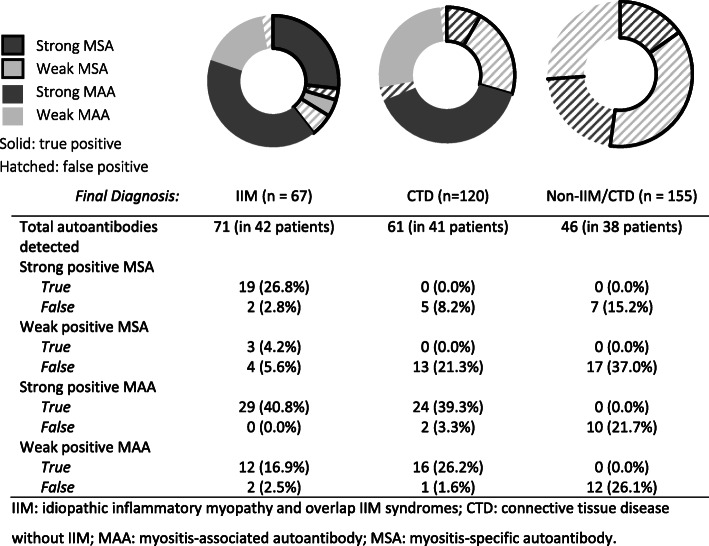
Categorisation of autoantibodies by final diagnosis, subtype, and strength of result

At least 1 Ab was detected in 42/67 (62.7%) IIM patients (Table 2). Their clinical phenotypes included DM (*n* = 11), IMNM (*n* = 11), ASS (*n* = 7), clinically amyopathic dermatomyositis (*n* = 4), PM (*n* = 4), and IBM (*n* = 2). In these 42 patients, a total of 71 Abs were detected (21 patients had multiple Abs; Fig. 2). 50/71 (70.4%) Abs were strongly positive (21 MSAs, 29 MAAs), and the majority of these were true positives (48/50 [96.0%]). 2/50 strongly positive Abs were false, and both were MSAs (anti-PL7 and anti-SAE1). Both patients had ASS and each had another concurrently detected MSA that was deemed to be the true positive. There were 21/71 (29.6%) weak positive Abs (7 MSAs, 14 MAAs). Of these, 15/21 (71.4%) were true positives (3 MSAs, 12 MAAs) and 6/21 (28.6%) were false positives (4 MSA, 2 MAAs).

**Table 2:** Cases of antibody positivity by final diagnosis

IIM	42/67 (62.7%)
Only weak MSA/MAA	9 (13.4%)
Only strong MSA/MAA	27 (40.3%)
Both weak and strong MSA/MAA	6 (9.0%)
CTD without IIM	41/120 (34.2%)
Only weak MSA/MAA	13 (10.8%)
Only strong MSA/MAA	18 (15.0%)
Both weak and strong MSA/MAA	10 (8.3%)
Non-IIM/CTD	38/155 (24.5%)
Only weak MSA/MAA	22 (14.2%)
Only strong MSA/MAA	13 (8.4%)
Both weak and strong MSA/MAA	3 (1.9%)
Any positive MSA/MAA across all diagnostic groups	121 (35.4%)

*IIM* idiopathic inflammatory myopathy, *CTD* connective tissue disease, *MSA* myositis-specific autoantibody, *MAA* myositis-associated autoantibody

Seven IIM patients had apparent dual MSA positivity. 3/7 (43.0%; DM (*n* = 2) and OM (*n* = 1)) had concurrent anti-Mi2A and anti-Mi2B. 1/7 (14.3%; ASS (*n* = 1)) was strongly positive for both anti-EJ and anti-PL7; anti-EJ was deemed the true positive based on significantly higher signal intensity when reviewed with the source data compared to anti-PL7 which just met the cut off for a strong positive result. Another was strongly positive for both anti-Jo1 and anti-SAE1; anti-Jo1 was felt to be the true positive as the patient clinically had ASS. 2/7 (28.6%) patients had dual weak false positives (anti-MDA5 and anti-TIF1γ in a patient with OM; anti-SAE1 and anti-SRP in a patient with PM).

### Autoantibody profiles in CTD patients without myositis: *False positive MSAs are detected commonly*

At least 1 Ab was detected in 41/120 (34.2%) patients with CTD without myositis (Fig. 2 and Table 2). Systemic sclerosis was the most common diagnosis. A total of 61 Abs were detected these 41 individuals with 31/61 (50.8%) being strongly positive; 24/31 (77.4%) of these were true strong positives, and all were MAAs (anti-Ro52 [*n* = 20], anti-PMScl100 [*n* = 1], anti-PMScl75 [*n* = 1], anti-Ku [*n* = 2]). Of the strong false positive Abs (*n* = 7), 5 were MSAs (*n* = 1 for each of anti-EJ, anti-Mi2A, anti-SAE1, anti-SRP, anti-PL12), and 2 were MAAs (anti-Ku in a patient with resolved undifferentiated connective tissue disease and anti-PM-SCL75 in a patient with systemic lupus erythematosus).

Weakly positive Abs were more common in CTD patients without myositis (30/61 [49.1%]; 13 MSAs, 17 MAAs) compared to IIM patients (21/71 [29.6%]; 7 MSAs, 14 MAAs; *p* = 0.02). 16/30 (53.3%) were true positives (all MAAs), and of the remaining false positives, 1 MAA and 13 MSA were detected (anti-SRP [*n* = 4], anti-PL12 [*n* = 2], anti-PL7 [*n* = 2], anti-TIF1*γ* [*n* = 2], anti-Jo [*n* = 1], anti-Mi2A [*n* = 1], and anti-OJ [*n* = 1]).

Four patients in the CTD group without myositis had multiple MSAs. All were false positives, and all had an anti-SRP (anti-SRP with anti-OJ, anti-SRP with anti-PL12, anti-SRP with anti-EJ, and anti-SRP with both anti-Mi2A anti-PL12).

### Autoantibody profiles in non-IIM/CTD patients: *Detection of MSAs and MAAs is common*

At least 1 Ab was detected in 38/155 (24.5%) non-IIM/CTD patients with a total of 46 detected Abs in these 38 individuals (24 MSAs [52.2%], 22 MAAs [47.8%]) (Fig. 2 and Table 2). All were deemed to be false positive results. 17/46 (37.0%) Abs were strong positives and 7/17 (41.2%) of these were MSAs. The final diagnoses for the 6 patients with strongly positive MSAs deemed to be false positives included immune checkpoint inhibitor related myofasciitis (*n* = 1; anti-EJ), genetic myopathy (*n* = 1; anti-Mi2B with anti-PL7), fibromyalgia (*n* = 1; anti-Jo1), and unclear final diagnoses (*n* = 3; anti-Mi2B, anti-TIF1γ, and anti-EJ).

29/46 (63.0%) Abs were weakly positive and 17/29 of these were MSAs. In these, the final diagnoses were immune checkpoint inhibitor related myofasciitis (*n* = 1; anti-SRP), primary Raynaud’s phenomenon (*n* = 1; anti-TIF1*γ*), fibromyalgia (*n* = 1; anti-Mi2B), statin-related toxic myopathy (*n* = 1; anti-Mi2B with anti-SAE1), polymyalgia rheumatica (*n* = 2; anti-OJ, anti-PL7), familial amyloidosis (*n* = 1; anti-SAE1), and unclear (*n* = 8; anti-SRP [*n* = 3], anti-TIF1γ [*n* = 2], antiPL7 with anti-SRP [*n* = 1], anti-Mi2A with anti-SRP [*n* = 1], anti-PL12 with anti-SRP [*n* = 1]).

Apparent dual positive MSAs were seen in 4 cases: anti-SRP with anti-PL12, anti-SRP with anti-Mi2B, anti-Mi2B with anti-SAE1, and anti-PL7 with anti-Mi2B.

### Factors associated with true and false positive antibody results

#### Weakly positive MSAs are often false positives

Across all diagnostic groups, weak positive MSAs were more likely to be false positives than true positives (34/37 [91.9%] false positives vs. 3/37 [8.1%] true positives), although the same was not true for weak positive MAAs (15/43 [34.9%] false positives vs. 28/43 [65.1%] true positives). This contrasts with strongly positive results, where the majority were true positives (19/33 [57.6%] true positives for MSAs, 53/65 [81.5%] for MAAs).

All weak positive anti-SRP (*n* = 10), anti-TIF1*γ* (*n* = 6), anti-SAE1 (*n* = 3), anti-PL7 (*n* = 3), anti-OJ (*n* = 2), anti-Mi2B (*n* = 2), anti-Jo1 (*n* = 1), and anti-MDA5 (*n* = 1) were false positives. For anti-PL12, false weak positivity was found in 4/5 (80.0%) cases. Amongst MAAs, false weak positives were found in 3/4 (75.0%) cases of anti-Ku and 6/14 (42.9%) cases of anti-Ro52. Anti-PM-Scl100 and anti-PM-Scl75 appeared to perform better with false weak positives only in 1/7 (14.3%) cases and 5/18 (27.8%) cases, respectively.

#### Patterns of anti-PM-Scl100 and − 75 positivity

There were 7 patients with dual positivity for anti-PM-Scl100 and anti-PM-Scl75, and all were true positives. Three cases had dual weak positive results and 4 had dual strong positive results. Four of these cases occurred in IIM patients with OM (scleromyositis) and 1 with amyopathic DM. The remaining 2 occurred in systemic sclerosis patients without myositis. Anti-PM-Scl100 occurred independently of anti-PM-Scl75 in 5 cases, 4 of which (80.0%) were true positives. Anti-PM-Scl75, however, occurred independent of anti-PM-Scl100 in 19 cases where 12/19 (63.2%) were true positives.

#### Sensitivity, specificity, and factors associated with a positive result

When considering individual Abs, specificity for the presence of a consistent diagnosis or disease subtype was generally high across all Abs (98.2–100.0% for weak positives and 97.5–100.0% for strong positives; Supplementary Table 1). However, weak positive anti-SRP had the lowest specificity (97.0%). A pre-test working diagnosis of IIM and a strong positive Ab result were significantly associated with true positivity (OR 50.8, 95%CI 13.66–189.22, *p* < 0.001 and OR 4.38, 95%CI 2.32–8.26, p < 0.001, respectively) (Table 3).

**Table 3:** Associations between true positive myositis-specific and myositis-associated autoantibodies and clinical factors

Factor	Any true positive MSA/MAA	Any false positive MSA/MAA	OR	P	CI
Female gender	78% (80/103)	67% (50/75)	1.74	0.10	0.89–3.39
Mean age of onset (SD)	50 (13)	52 (15)	0.99	0.35	0.96–1.01
Pre-test working diagnosis of IIM	59% (61/103)	19% (14/75)	50.8	**<0.001**	13.66–189.22
Biopsy in keeping with inflammatory myopathy	50% (7/14)	28% (7/25)	2.57	0.18	0.66–10.06
Myopathic EMG changes	64% (21/33)	43% (12/28)	2.33	0.11	0.83–6.54
Highest recorded CK (mean, SD)	1377 (3502)	563 (910)	1.0	0.108	1.00–1.00
Strong positive antibody result	70% (72/103)	35% 26/75	4.38	**<0.001**	2.32–8.26

*MSA* myositis-specific autoantibody, *MAA* myositis-associated autoantibody, *OR* odds ratio, *P* p-value, *CI* confidence intervals, *SD* standard deviation, *IIM* idiopathic inflammatory myopathy, *EMG* electromyography, *CK* creatine kinase

## Discussion

This study evaluated the clinical performance of the EUROIMMUN Inflammatory Myopathies 16 Ag LIA, a commonly used commercial assay. It is also the largest to report factors associated with true positive results. We showed that in expert-diagnosed IIM cases, 62.7% of patients had at least 1 identified Ab on LIA. A strong positive result and a high pre-test diagnosis of IIM was most associated with true positive results. This emphasises the value of an expert clinician’s initial impression in reaching an IIM diagnosis and that LIA testing for IIM should be applied with caution in patients with low diagnostic suspicion for IIM.

A weak positive MSA was much more likely to be a false positive across all diagnostic groups (34/37 [91.9%] false positives vs. 3/37 [8.1%] true positives). This was particularly true for weakly positive anti-SRP results which were all false positives in our study. Our study is in agreement with other recent publications which have also demonstrated that weak positives are more likely to be false [24]. Whilst weak positive MSAs were more likely to be false positives, specificity was high. Weak positive anti-SRP had the lowest specificity. Our results suggest that this LIA’s accuracy may be improved if the threshold for defining weak positivity was increased, although this may vary according to each antibody on the assay [25].

We also found only 4/67 (5.9%) IIM cases with multiple MSAs (excludes concurrent anti-Mi2A and anti-Mi2B, isoforms of Mi2 autoantibodies which co-exist frequently [23]). Two of these cases only had 1 true MSA each and in the other 2 cases, all were false positive results. This is congruent with recent large cohort studies demonstrating mutual exclusivity of MSAs in IIM individuals [8] and highlights that when multiple MSAs are found in LIA testing, results should be treated with suspicion. Dual positivity for the MAAs anti-PM-Scl100 and anti-PM-Scl75, in contrast, improved the reliability of the results in both IIM and CTD without IIM cases.

Another notable finding was that a high proportion of IIM patients were seronegative (37.3%). This number is comparable to recent findings from a large cohort of European IIM patients which found 38.3% of their cases to be seronegative [8]. A growing number of Abs currently not available in this LIA may be useful in the correct clinical context. For example, in addition to anti-HMGCR, recent larger cohorts of IIM demonstrate that other emerging Abs such as anti-KS and anti-Zo are also useful in the diagnosis of IIM [8] .

Limitations of this study include data drawn from a single centre, although they represent a population of nearly 3 million people. Secondly, data were analysed retrospectively, and no specific additional review or tests were performed to confirm the diagnostic categorisation. Of the patients without CTD or IIM, most had at least 3 years of follow-up in their case notes, but it remains possible that positive Ab results may represent preclinical IIM or CTD. Additionally, negative Ab results were assumed to be true negatives. It is possible that some seronegative patients have detectable Abs via another method such as immunoprecipitation or have a hitherto undescribed Ab. Additionally, in cases where duplicate testing on the same patient occurred, we included only the most recent results. There is some evidence that certain MSAs might be lowered with treatment [26] so including only the most recent LIA result may have affected our results. However, clinicians seldom use the LIA for disease monitoring and the seven duplicates which occurred were more likely to be cases where the accuracy of first LIA test was in question. Finally, the final diagnoses made by the treating physicians could have been biased by the Ab results. However, most patients had several years of follow-up allowing for their diagnoses to be confirmed or reclassified over time and these final diagnoses were used in this study.

## Conclusions

MSAs and MAAs are increasingly gaining importance in the diagnostic workup and management of IIMs. Clinicians may rely on commercially available LIAs to identify these Abs. This study describes the clinical utility of a widely used LIA and is the largest to report factors associated with true positive results. We demonstrated that strongly positive autoantibodies and a high pre-test likelihood of IIM are associated with true positive results on LIA. Specificity for individual MSAs on the LIA for IIM is high. Weak positive MSA results are more likely to be false than true positives. Our data suggests that cut-off values of the LIA may need to be redefined to increase its accuracy. Our work adds to the understanding of the increasing role of LIA testing for MSAs and MAAs in routine clinical practice and will support clinicians in understanding the relevance and implications of results.

## Supplementary information

**Additional file 1: Table S1.** Myositis specific and associated autoantibodies specificities and categorisation by strength of positivity and true/false positive rate.

## Data Availability

The datasets generated and/or analysed during the current study are available in the electronic health records employed by the Salford Royal NHS Foundation Trust.

## Abbreviations

Ab: Autoantibody
ASS: Anti-synthetase syndrome
CK: Creatine kinase
CTD: Connective tissue disease
DM: Dermatomyositis
EMG: Electromyography
IBM: Sporadic inclusion-body myositis
IIM: Idiopathic inflammatory myopathy
IMNM: Immune-mediated necrotising myopathy
LIA: Line blot immunoassay
MAA: Myositis associated autoantibody
MSA: Myositis specific autoantibody
OM: Overlap myositis
PM: Polymyositis
SRFT: Salford Royal NHS Foundation Trust
