# Supplementary material for: Line blot immunoassays in idiopathic inflammatory myopathies: retrospective review of diagnostic accuracy and factors predicting true positive results
Source: BMC Rheumatol. 2020 Jul 20;4:28. doi: 10.1186/s41927-020-00132-9 (PMC7370419; doi:10.1186/s41927-020-00132-9)
Supplement: Supplementary file 1 — Additional file 1: Table S1. Myositis specific and associated autoantibodies specificities and categorisation by strength of positivity and true/false positive rate. [file 41927_2020_132_MOESM1_ESM.docx]

# Supplementary table

**Supplementary Table 1. Myositis specific and associated autoantibodies specificities and categorisation by strength of positivity and true/false positive rate.**

|  |  | WEAK POSITIVE | | | STRONG POSITIVE | | |  |
| --- | --- | --- | --- | --- | --- | --- | --- | --- |
| **Antibody** |  | FALSE (%) | TRUE (%) | Specificity** | FALSE (%) | TRUE (%) | Specificity** | **Total** |
|  | Negative (%)* |  |  |  |  |  |  |  |
|  |  |  |  |  |  |  |  |  |
| **EJ** | 339 (99.1) | 0 | 0 | 100 | 2 (0.6) | 1 (0.3) | 99.4 | **342** |
| **JO1** | 336 (98.2) | 1 (0.3) | 0 | 99.7 | 1 (0.3) | 4 (1.2) | 99.7 | **342** |
| **KU** | 333 (97.4) | 3 (0.9) | 1 (0.3) | 99.1 | 3 (0.9) | 2 (0.6) | 99.1 | **342** |
| **MDA5** | 341 (99.7) | 1 (0.3) | 0 | 99.7 | 0 | 0 | 100 | **342** |
| **MI2A** | 336 (98.2) | 2 (0.6) | 2 (0.6) | 99.4 | 1 (0.3) | 1 (0.3) | 99.7 | **342** |
| **MI2B** | 334 (97.7) | 2 (0.6) | 0 | 99.4 | 3 (0.9) | 3 (0.9) | 99.1 | **342** |
| **NXP2** | 341 (99.7) | 0 | 0 | 100 | 0 | 1 (0.3) | 100 | **342** |
| **OJ** | 340 (99.4) | 2 (0.6) | 0 | 99.4 | 0 | 0 | 100 | **342** |
| **PL12** | 335 (98.0) | 4 (1.2) | 1 (0.3) | 98.8 | 1 (0.3) | 1 (0.3) | 99.7 | **342** |
| **PL7** | 337 (98.3) | 3 (0.9) | 0 | 99.1 | 2 (0.6) | 0 | 99.4 | **342** |
| **RO52** | 281 (82.2) | 6 (1.8) | 8 (2.3) | 97.9 | 7 (2.0) | 40 (11.7) | 97.5 | **342** |
| **SAE1** | 332 (97.1) | 3 (0.9) | 0 | 99.1 | 2 (0.6) | 5 (1.5) | 99.4 | **342** |
| **SCL100** | 330 (96.5) | 1 (0.3) | 6 (1.8) | 99.6 | 0 | 5 (1.5) | 100 | **342** |
| **SCL75** | 316 (92.4) | 5 (1.5) | 13 (3.8) | 98.4 | 2 (0.6) | 6 (1.8) | 99.3 | **342** |
| **SRP** | 331 (96.8) | 10 (2.9) | 0 | 97 | 1 (0.3) | 0 | 99.6 | **342** |
| **TIF1G** | 332 (97.1) | 6 (1.8) | 0 | 98.2 | 1 (0.3) | 3 (0.9) | 99.6 | **342** |
| ***Any MSA*** | *4034 (98.3)* | *34 (0.8)* | *3 (0.1)* | *99.2* | *14 (0.3)* | *19 (0.5)* | *99.7* | ***4104*** |
| ***Any MAA*** | *1260 (92)* | *15 (1.1)* | *28 (2.1)* | *98.8* | *12 (0.9)* | *53 (3.9)* | *99.1* | ***1368*** |
| ***Totals*** | *5294 (96.7)* | *49 (0.9)* | *31 (0.6)* | *99* | *26 (0.5)* | *72 (1.3)* | *99.5* | ***5,472*** |

All final diagnoses considered. *all negative assumed to be true negative. Sensitivity was not calculated. **Specificity refers to the presence of a consistent diagnosis or disease subtype.
